# Phenotypic variability in a child with Felty’s syndrome: a case report

**DOI:** 10.1186/s12887-020-02054-4

**Published:** 2020-04-07

**Authors:** Guo-min Li, Hai-mei Liu, Wan-zhen Guan, Yi-fan Li, Hong Xu, Li Sun

**Affiliations:** Department of Rheumatology, Children’s Hospital of Fudan University, 399 Wan-yuan Road, Shanghai, 201102 China

**Keywords:** Children, Felty’s syndrome, Juvenile idiopathic arthritis, Thrombocytopenia

## Abstract

**Background:**

Felty’s syndrome (FS) is characterized by the triad of rheumatoid arthritis (RA), splenomegaly and neutropenia. The arthritis is typically severe and virtually always associated with high-titer rheumatoid factor. The presence of persistent neutropenia is generally required to make the diagnosis. Most patients diagnosed with FS are aged 50–70 years and have had RA for more than 10 years. It is rarely seen in patients with juvenile idiopathic arthritis (JIA), with only five cases having been reported throughout the world.

**Case presentation:**

The present study describes the case of a 14-year-old female with a seven-year history of polyarticular JIA, presenting with splenomegaly, hepatomegaly, cholestasis and thrombocytopenia. However, she occasionally developed neutropenia. Titers of rheumatoid factor and anti-CCP were persistently high, and the antinuclear antibody titer was 1:320, while the antibody results for anti-dsDNA and anti-Sm were negative. Serum levels of IgA, IgG, IgM and IgE were all persistently elevated, and the ratio of CD19^+^ lymphocytes in the subgroups of lymphocytes was persistently high. The level of complements was normal. No STAT3 and STAT5B mutations were found by next-generation sequencing. The patient did not respond to methotrexate, prednisolone, hydroxychloroquine (HCQ), sulfasalazine and etanercept but was responsive to rituximab.

**Conclusions:**

JIA, thrombocytopenia and splenomegaly are the most common and important features in six children with FS, while persistent neutropenia is not seen in all these patients. No complement deficiency has been found in children with FS so far. Manifestations of FS without neutropenia may be extremely rare. There are differences between adults and children in the clinical and laboratory features of FS.

## Background

Felty’s syndrome (FS) was first described in 1924 at Johns Hopkins hospital by the American physician, Augustus Roi Felty [1]. The term was first used by Hanrahan and Miller in 1932 when they described the beneficial effect of splenectomy in a patient with features similar to the five cases reported by Felty. It is a specific subcategory of rheumatoid arthritis (RA) characterized by the triad of seropositive RA with severe joint involvement, splenomegaly and neutropenia. Typically, FS tends to affect patients with long-standing erosive RA. It usually develops after a > 10-year course of RA [2], with increased risk in patients with a positive family history of RA. The complete triad is not an absolute requirement, but persistent neutropenia with an absolute neutrophil count (ANC) less than 1500–2000/mm^3^ is generally regarded as necessary for establishing the diagnosis in adult [3]. FS affects 1–3% of patients with RA, but it is rarely seen in patients with juvenile idiopathic arthritis (JIA), with only a few cases having been reported throughout the world [4–8]. The active extra-joint clinical features can be misleading in FS, and certain pediatrician focus on severe extra-articular disease and neutropenia, which suggests infectious diseases. Thus, the correct diagnosis is occasionally challenging for children rheumatologist. We describe an additional case of a child with FS, compare it to the previous reports, and discuss differences between adults and children in clinical and laboratory features of FS through a literature review, in order to better understand the disease.

## Case presentation

**Fig. 1:**
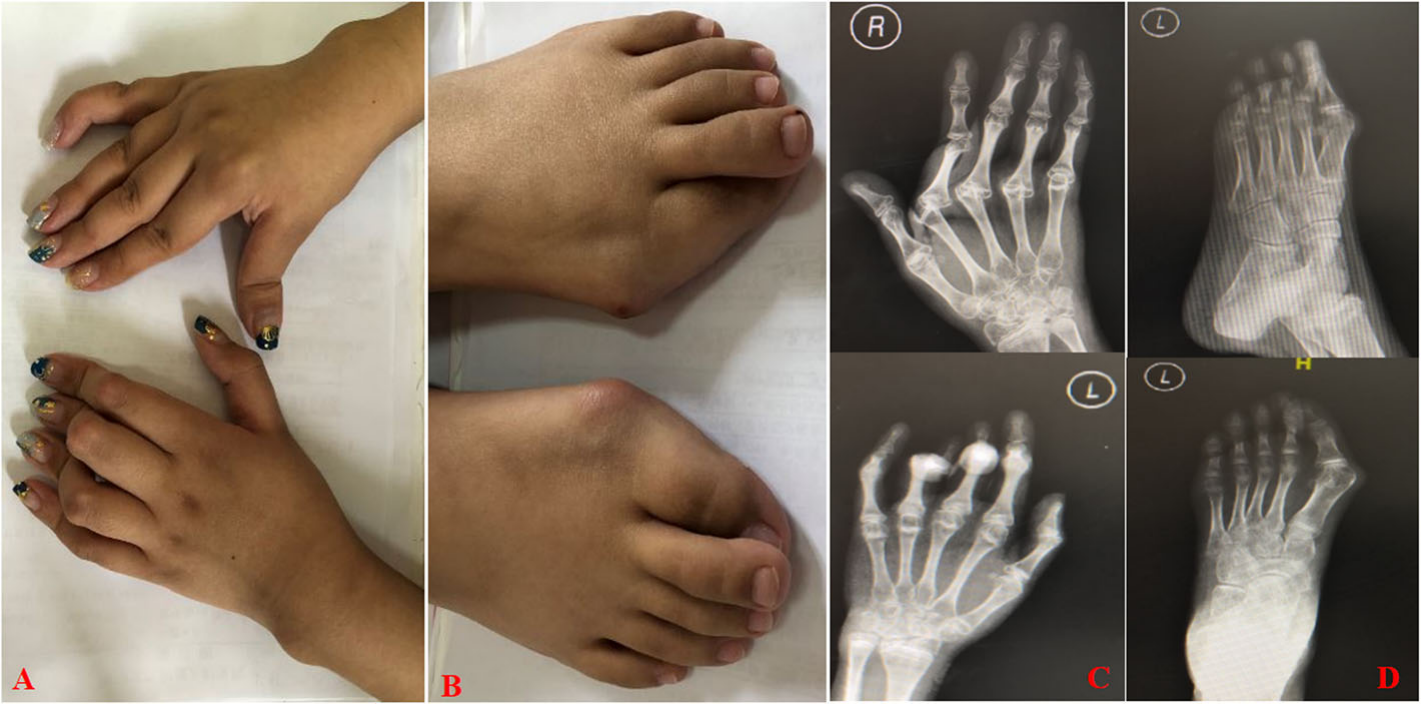
**a** shows deformities in the proximal interphalangeal joints and wrists, and **b** shows deformities in the metacarpophalangeal joints and ankles; **c** reveals deformities of left and right hands, and **d** reveals deformities of left foot form positive and lateral position by X-rays

**Fig. 2:**
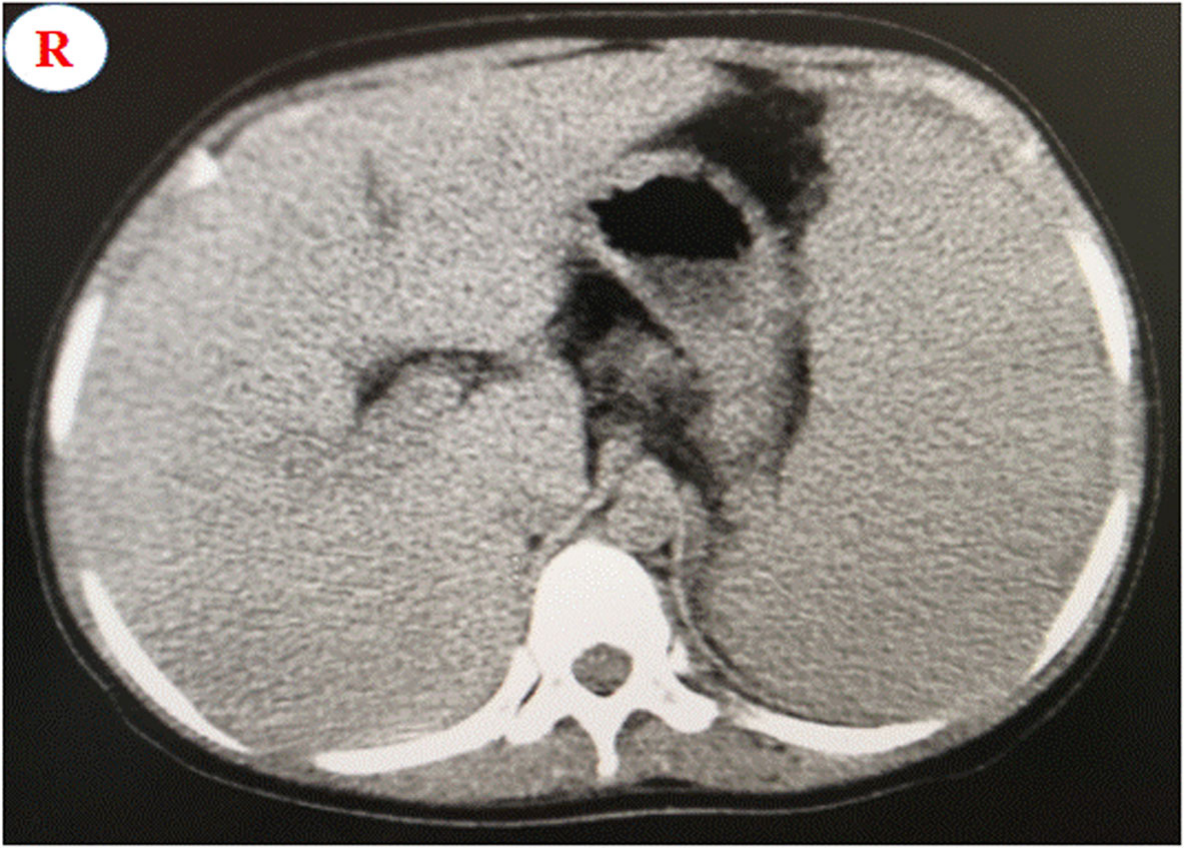
Abdominal contrast-enhanced CT revealed splenomegaly

The patient, an 8-year-old Chinese girl, presented with pain of the left hip in Jan 2011, which resolved spontaneously over several weeks. Seven months later, she presented with pain of the left hip again. She was diagnosed with JIA because of the chronic synovitis of the left hip joint at the local hospital in Jan 2012. She had high titers of RF and anti-CCP, while her complete blood count was normal at that time. Clinical examination revealed splenomegaly. Treatment was initiated with diclofenac sodium (2–3 mg/kg/day), methotrexate (10–15 mg/m^2^/week) and hydroxychloroquine (5 mg/kg/day). Her symptoms improved within 3 months, and treatment was stopped by her parents in Oct 2012. However, she presented with pain and swelling in the hips, knees, ankles and wrists in Jan 2013. In addition, she gradually developed morning stiffness and synovitis of proximal interphalangeal joints and metacarpophalangeal joints. An abdominal ultrasound revealed hepatomegaly and splenomegaly. She was given oral prednisolone (1 mg/kg/day) combined with diclofenac sodium, methotrexate and hydroxychloroquine. Within several months, the patient had almost no pain or stiffness and minimal joint swelling on examination. However, the proximal interphalangeal and metacarpophalangeal joints gradually developed deformities. Complete blood counts were obtained every 4 to 6 weeks over the next year and showed persistent thrombocytopenia (range from 48 to 94 × 10^9^/L) but no neutropenia. She received etanercept (0.8 mg/kg/week) treatment in Jan 2014 due to progressive aggravation of joint deformity. She was admitted to our hospital for further assessment due to the chronic synovitis of her joints, hepatosplenomegaly and thrombocytopenia in Jul 2015. On admission, blood tests revealed a white blood cell count of 6.2 × 10^9^/L with an absolute neutrophil count of 3.92 × 10^9^/L, hemoglobin levels of 118 g/L and a platelet count of 95 × 10^9^/L. The peripheral blood smear was normal. A bone marrow aspirate showed normal trilineage hematopoiesis with no infiltration malignant cells or large granular lymphocytes. Levels of glutamic oxaloacetic transaminase and glutamate pyruvate transaminase were normal, while total bilirubin levels were 43.1 μmol/L (normal range 3.4–17.1), direct bilirubin levels were 18.7 μmol/L (normal range 0–6) and total bile acid levels were 203.0 μmol/L (normal range0–10). A rapid erythrocyte sedimentation rate (ESR, 35 mm/h) and high C-reactive protein levels (CRP, 60.2 mg/l) were revealed. Tests for human immunodeficiency virus, syphilis, hepatitis B, hepatitis C and autoimmune hepatitis-associated antibodies and a purified protein derivative skin test produced negative findings. The antinuclear antibody titer was 1:100, while the results for anti-dsDNA, anti-Sm and anti-Rnp antibodies were negative. Levels of complement C3 and C4 were normal. Serum levels of IgA were 2.97 g/L, IgG15.49 g/L, IgM 2.39 g/L and IgE 319.1 g/L and were all elevated. Levels of rheumatoid factor and antibodies to cyclic citrullinated peptides were 10,200.0 U/L and 384.0 U/L, **r**espectively. X-rays of the hand joints showed soft tissue swelling, bone erosion and narrowing of the joint cavity. A physical examination revealed splenomegaly, hepatomegaly and deformities in the proximal interphalangeal joints, metacarpophalangeal joints, wrists and ankles (Fig. 1a and b), which were confirmed by X-rays and computed tomography (Fig. 1 c, d and Fig. 2), respectively. Examination of the other systems was unremarkable, such as no peripheral and central lymph nodes of enlargement. Hydroxychloroquine and etanercept were stopped, prednisolone and methotrexate were maintained at 15 mg daily and 15 mg weekly, respectively, and sulfasalazine was added at 20 mg/kg daily. Treatment with rituximab was started twice at 375 mg/m^2^ over 2 weeks in July 2015 and then once at 375 mg/m^2^ every 6 months. One month after the start of treatment, CRP decreased from 60.2 mg/L to 6 mg/L, and the ESR decreased from 35 mm/h to 8 mm/h. Over 6 months, the prednisone was tapered to her current dose of 7.5 mg/day.

**Fig. 3:**
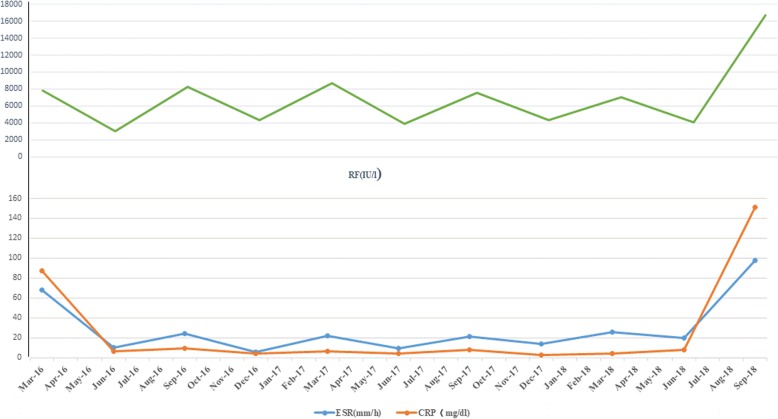
Following-up of ESR, CRP and RF after treatment with rituximab. They fell transiently to a normal range after treatment and rose before the next treatment

The results of previous clinical and laboratory evaluations were obtained from earlier outpatient records. Pertinent results from the last three consecutive years are shown in Fig. 3. Since the onset of her arthritis, the total white blood cell (WBC) count was normal and occasionally below 4.0 × 10^9^/L. Regular follow-up visits were scheduled for her after Jan 2016. Although her ESR, CRP and count of CD19^+^ lymphocytes fell transiently to the normal range after treatment with rituximab, they rose before the next treatment (see Fig. 3). Titers of RF (see Fig. 3) and levels of all immunoglobulins (see Table 1) were persistently high. The ratio of CD19+ lymphocytes in subgroups of lymphocytes were persistently high, while the count of CD16^+^CD56^+^ lymphocytes was persistently below the normal range. She experienced a fever, rising ESR and CRP, increasing RF (68,000 U/L) and anti-CCP (320 U/L) titers, and falling WBC and platelet count in July 2018. However, she had no hyperferritinemia, hypertriglyceridemia, hypofibrinogenemia. Rituximab 375 mg/m^2^ every 6 months was adjusted to the same dosage every 4 months. Since then, her neutrophil count, platelet count, ESR and CRP have been in a persistently normal range. Next-generation sequencing was performed in July 2018 because she presented with refractory FS. No STAT3 or STAT5B mutations were found.

**Table 1:** Immunoglobulin subgroups

Immunoglobulin	2016(y)			2017(y)				2018(y)			Normal range
	04/08	23/09	01/12	13/07	28/08	09/10	16/11	01/03	10/09	27/09	
IgA(g/L)	2.86	2.97	1.76	3.38	4.18	3.55	2.88	5.01	6.34	5.45	0.52–2.16
IgG(g/L)	14.41	15.49	11.62	14.60	16.0	16.1	14.4	15.4	20.8	17.0	6.09–12.85
IgM(g/L)	2.21	2.39	1.09	4.39	5.89	3.81	3.19	7.39	9.45	7.25	0.67–2.01
IgE (KU/L)	112.6	319.1	71.7	141.36	346.7	147.2	314.48	269	4280.14	1949.12	< 100

## Discussion and conclusion

The PubMed database was searched for all publications with the keywords or MeSH terms “Felty’s syndrome” AND “pediatric” or “child”. Only English language articles were included. We also searched the Chinese Journal Full-text Database (CNKI) using the same keywords in Chinese. A few case studies for children with FS have been published in English, but no Chinese studies were found. We performed a literature review and identified 5 children with FS [4–8]. Table 2 shows the major features of these cases. Six children with FS have been identified, and all of these cases are female. The average age at onset of JIA is only 9.2 years (range from 4.0–15.0), and the duration of JIA until FS is 6.2 years (range from 4.0–15.0).

**Table 2:** clinical features of six children with FS

Clinical features	Case 1	Case 2	Case 3	Case 4	Case 5	Case 6
Age at onset of JIA(Y)	10.0	4.0	4.0	14.0	15.0	8.0
Gender	F	F	F	F	F	F
JIA subtype	Polyarticular	Polyarticular	Polyarticular	Systemic	Polyarticular	Polyarticular
Duration of JIA until FS(Y)	5.8	11.0	4.5	10.0	2.0	4.0
Splenomegaly	Yes	Yes	Yes	Yes	Yes	Yes
Hepatomegaly	No	No	No	Yes	Yes	Yes
Hemoglobin(g/l)	Normal	135.0	122.0	128.0	80.0	118.0
Platelet count (×10^9^/l)	Not reported	313.0	72.0	105.0	159.0	95.0
Leucocyte count (×10^9^/l)	1.8	4.1	2.8	2.7	4.1	6.2
Granulocyte count (× 10^9^/l)	0.5	1.7	1.3	1.5	3.1	3.92
RF (IU/l)	1:480 Positive	Negative	Negative	Negative	Negative	10,200.0 Positive
Anti-CCP(U/l)	Not reported	Not reported	Not reported	Not reported	Negative	384.0
Levels of complement	Normal	NA	Normal	NA	Normal	Normal
ANA	1:120 Positive	1:320 Positive	Negative	Negative	Negative	1:320 Positive
Bone marrow findings	Normal	Normal	Normal	Normal	Normal	Normal
Medications	MTX, Ibuprofen	Gold, HCQ, ASA	Tolmetin, ASA	Prednisolone, HCQ, Ibuprofen, MTX	MTX, HCQ, MP	Prednisolone, Diclofenac, HCQ, Sulfasalazine, MTX, Etanercept, Rituximab
Reference	[4]	[5]	[6]	[7]	[8]	*

*MTX* Methotrexate, *HCQ* Hydroxychloroquine, *ASA* Acetylsalicylic acid, *MP* Methylprednisolone; *, our patient

FS is an uncommon but severe extra-articular manifestation of rheumatoid arthritis, including hepatopathy, lymphadenopathy, vasculitis, leg ulcers, abnormal skin pigmentation and a high frequency of rheumatoid nodules [2, 9]. There is no specific diagnostic criterion for FS. FS is a clinical diagnosis in patients with RA or JIA with unexplained neutropenia and splenomegaly [2, 10].

Although the patient in our study presented with hip arthritis, she gradually developed morning stiffness and synovitis of proximal interphalangeal joints and metacarpophalangeal joints. Not only that, she had high RF and anti-CCP titers. Thus, she fulfills polyarthritis (rheumatoid factor positive) of 2001 ILAR juvenile idiopathic arthritis classification [11]. In addition, she had splenomegaly, neutropenia and thrombocytopenia. Bone marrow aspirate and peripheral blood smear ruled out large granular lymphocyte syndrome, hematological neoplasm, and suppression of hematopoiesis by medications (such as methotrexate). Therefore, she met the diagnosis criteria of FS. However, our patient presented with occasional neutropenia rather than persistent neutropenia. Recurrent thrombocytopenia was more common than occasional neutropenia in the patient. Some laboratory features of our patient overlap with systemic lupus erythematosus (SLE), such as neutropenia, thrombocytopenia and positive ANA. However, the ANA titer was low (1:320), and the results of anti-dsDNA and anti-Sm antibodies were negative, which did not support the diagnosis of SLE. Although she experienced a fever, falling WBC and platelet count, and splenomegaly, she had no hyperferritinemia, hypertriglyceridemia, hypofibrinogenemia. In addition, no hemophagocytic cells were found by bone marrow aspiration. Therefore, she was not diagnosed with macrophage activation syndrome (MAS).

Current data show that 1–3% of RA patients are complicated with FS, with an estimated prevalence of 10 per 100,000 populations [12]. FS is rarely seen in patients with JIA, with only five cases having been reported throughout the world [4–8]. Table 2 provides a comparison of these five patients with our patient (patient 6). The six patients were all female. Although patient 4 had arthritis in the adolescent period, she developed FS in the adult period. Patient 6 and patient 1 developed seropositive (RF+) JIA, and the other four patients developed seronegative (RF-) JIA. Except for patient 4 with systemic JIA, patient 6 and the other four patients had polyarticular JIA. All six patients had splenomegaly, while patient 6, patient 1 and patient 2 had hepatomegaly. Patient 6 developed occasional neutropenia, which differed from other five patients, of which four had persistent neutropenia and one had no neutropenia. Patient 6, patient 3, and patient 4 all had thrombocytopenia. The level of hemoglobin was below the normal range only in patient 5.

Adult FS is three times more common in females [2], but most children with FS have been females so far. Adults diagnosed with FS are usually 50–70 years of age and have had RA for more than 10 years [9, 13], while the average age at onset of JIA is only 9.2 years (range from 4.0–15.0), and the duration of JIA until FS is 6.2 years (range from 4.0–15.0). Therefore, FS usually develops late in RA and JIA. Although FS is a severe form of RA, it can be asymptomatic. In very rare and adult cases, manifestations of FS had no clinical but only laboratory features of RA [13–16]. Therefore, joint involvement is not necessary for diagnosis and is absent in 15–40% of FS patients [2]. To date, FS without arthritis has not been reported in children. Splenomegaly is not always present in adult FS [8], but it occurs in all children with FS. Neutropenia is the most common and important feature of FS. Some authors consider all RA-associated neutropenia to be laboratory manifestations of FS [2, 9, 15, 17]. The complete triad is not an absolute requirement, but persistent neutropenia is necessary for establishing the diagnosis [3]. Of the six children with FS, one had no neutropenia, one had transient neutropenia, and the others had persistent neutropenia; thus, persistent neutropenia is not necessary for diagnosis in children. Thrombocytopenia is a common manifestation in children with FS, yet it seldom occurs in adults [2] (see Table 3). High titers of rheumatoid factor are present in most adult patients. Of the 72 patients with FS reviewed by Spivak, 94% had positive test results for rheumatoid factor and 63% had antinuclear antibodies [18], while 2 out of 6 children with FS had positive test results for rheumatoid factor and 3 out of 6 children had antinuclear antibodies. Complement components are usually depressed in adults with FS [2], but they are normal in children. Therefore, there are differences between adults and children in clinical and laboratory features of FS, and they are summarized in Table 3.

**Table 3:** Difference between adults and children in clinical and laboratory features of FS

Patient	F/M ratio	Arthritis	Splenomegaly	Leukopenia	Thrombocytopenia	Anemia	Hypocomplementemia	Autoantibody
Adults	2–3/1	common	common	Neutropenia (Always)	Rare	Present	Frequent	common
Children	All F	in all six	in all six	Neutropenia (Often)	Often	Present	None	three out of six

Although the precise pathophysiology of FS remains unknown, an autoimmune mechanism seems likely. The syndrome occurs almost exclusively in patients who have abnormal circulating autoantibodies, either rheumatoid factor, antinuclear antibodies, or both [19]. Our study showed that the levels of all immunoglobulin subgroups (see Table 1) and the ratio of CD19^+^ lymphocytes in subgroups of lymphocytes were persistently high in the patient, which supported that the autoimmune mechanism plays an important role in FS. Interestingly, all subgroups of immunoglobulin are elevated in patients, even immunoglobulin E (IgE). Raised levels of serum IgE and eosinophils are known as hallmarks in atopic patients. Anti-IgG antibodies (anti-IgG) of the IgE class, studied in sera from patients with JIA, in patients with RA and FS using an indirect immunofluorescence technique, showed IgE anti-IgG in 63% of patients with RA and in 80% of patients with FS [20]. The titers of IgE anti-IgG were significantly higher in the FS patients. Therefore, IgE anti-IgG may be regarded as part of a broad polyclonal antibody response to IgG molecules in FS patients [21].

There is no standard therapy for this disease, and several disease-modifying antirheumatic drugs have been used with varying success. MTX has been considered a first-line therapy for FS since Wassenberg et al. [22] reported the efficiency of a low dose in five of seven RA patients with FS. However, the MTX response was inconsistent, as it is in this case. Several alternative drugs – prednisolone, HCQ and etanercept – have been proposed [23, 24]. Unfortunately, our patient showed no response to prednisolone, HCQ and etanercept, as was previously observed [25, 26]. Rituximab, a chimeric anti-CD20 monoclonal antibody, is used in combination with methotrexate to treat active RA after failure or intolerance of at least one anti-TNF-α [27]. Therefore, HCQ and etanercept were switched to rituximab. Treatment with rituximab was started twice at 375 mg/m^2^ over 2 weeks and then once at 375 mg/m^2^ every 6 months. Although ESR and CRP rapidly decreased to the normal range after initial treatment with rituximab, they were elevated before the next treatment in the patient (see Fig. 3). Treatment with rituximab was adjusted to 375 mg/m^2^ every 4 months. The patient is being followed up closely. Until August 2019, her neutrophil count, platelet count, ESR and CRP have been in a persistently normal range, and joints had no swelling and pain.

Recently, somatic STAT3 and STAT5B mutations were discovered in 30–40% of patients with large granular lymphocyte leukemia [28, 29], and somatic STAT3 mutations were detected in 43% of FS patients with deep amplicon sequencing targeting all STAT3 exons [30]. Mutations were located in the SH2 domain of STAT3, which is a known mutational hotspot. We performed next-generation sequencing in the patient because she presented with refractory FS. However, no STAT3 mutations were found. This is concurrent with a study showing that in a cohort of 82 newly diagnosed RA patients, no STAT3 mutations were detected [31].

Here, we descripted FS girl without persistent neutropenia and low levels of complement. Manifestations of FS without neutropenia may be extremely rare. We only found six children with FS reported through literature review (including our patient), hence FS is rarely seen in children with JIA. JIA, thrombocytopenia and splenomegaly are the most common and important features in six children with FS, while persistent neutropenia is not seen in these patients. Low levels of complement are not found in any children with FS so far. There are differences between adults and children in the clinical and laboratory features of FS.

## Abbreviations

ANC: Absolute neutrophil count
CRP: C-reactive protein levels
ESR: Erythrocyte sedimentation rate
FS: Felty’s syndrome
HCQ: Hydroxychloroquine
JIA: Juvenile idiopathic arthritis
RF: Rheumatoid factor
RA: Rheumatoid arthritis
WBC: White blood cell
